# Management of Hemothorax After Blunt Chest Trauma: Results from a Level II Emergency Department

**DOI:** 10.3390/jcm15082814

**Published:** 2026-04-08

**Authors:** Dania Nachira, Antonio Giulio Napolitano, Adriana Nocera, Maria Teresa Congedo, Marcello Covino, Claudia Bellettati, Claudia Leoni, Chiara Scognamiglio, Giovanni Punzo, Mariano Alberto Pennisi, Nicola Bonadia, Maria Letizia Vita, Leonardo Petracca-Ciavarella, Filippo Lococo, Elisa Meacci, Stefano Margaritora

**Affiliations:** 1 Department of General Thoracic Surgery, Fondazione Policlinico Universitario “A. Gemelli”, IRCCS, Università Cattolica del Sacro Cuore, 00168 Rome, Italy; dania.nachira@unicatt.it (D.N.); antoniogiulionapolitano@gmail.com (A.G.N.); mariateresa.congedo@policlinicogemelli.it (M.T.C.); claudia.bellettati01@icatt.it (C.B.); claudia.leoni01@icatt.it (C.L.); kiasco1998@gmail.com (C.S.); marialetizia.vita@policlinicogemelli.it (M.L.V.); leonardo.petraccaciavarella@policlinicogemelli.it (L.P.-C.); filippo.lococo@policlinicogemelli.it (F.L.); elisa.meacci@policlinicogemelli.it (E.M.); stefano.margaritora@policlinicogemelli.it (S.M.); 2 Emergency Department, Fondazione Policlinico Universitario “A. Gemelli”, IRCCS, 00168 Rome, Italy; marcello.covino@policlinicogemelli.it (M.C.); nicola.bonadia@policlinicogemelli.it (N.B.); 3 Department of Emergency, Fondazione Policlinico Universitario “A. Gemelli” IRCCS, Università Cattolica del Sacro Cuore, 00168 Rome, Italy; 4 Department of Anesthesiology and Intensive Care Medicine, Fondazione Policlinico Universitario “A. Gemelli”, IRCCS, Università Cattolica del Sacro Cuore, 00168 Rome, Italy; giovanni.punzo@policlinicogemelli.it (G.P.); marianoalberto.pennisi@policlinicogemelli.it (M.A.P.)

**Keywords:** blunt hemothorax, chest trauma, management, thoracic surgery

## Abstract

**Background:** Traumatic hemothorax is a common complication of blunt chest trauma and remains associated with significant morbidity and mortality. Although contrast-enhanced computed tomography (CT) is central to diagnosis, the optimal criteria for selecting patients who require invasive management versus conservative treatment remain unclear. This study aimed to evaluate the management strategies and clinical outcomes of traumatic hemothorax and to identify predictors of surgical intervention and postoperative complications. **Methods:** We conducted a retrospective, single-center cohort study including adult patients admitted to a Level II Emergency Department with hemothorax following blunt chest trauma between January 2019 and December 2024. Primary outcomes were the need for urgent chest drainage or surgery. Secondary outcomes included postoperative complications, length of hospital stay, and intensive care unit admission. Univariable and multivariable regression analyses were performed to identify factors associated with surgical intervention and complications. **Results:** Seventy-two patients were included (mean age 60.0 ± 20.5 years; 80.6% male). Rib fractures were the most common cause of hemothorax (61.1%). Chest tube placement was required in 70.8% of cases, and 31.9% underwent urgent surgical intervention. Active bleeding on contrast-enhanced CT was identified in 16.7% of patients and was the only independent predictor of urgent surgery (OR 3.85, 95% CI 1.07–13.88; *p* = 0.039). The initial volume of blood drained after chest tube insertion did not differ between surgically and non-surgically managed patients. Conservative management was successful in 19.4% of cases. Postoperative complications occurred in five patients and were associated with a higher comorbidity burden. Overall mortality was 5.6%. **Conclusions**: In traumatic hemothorax following blunt chest trauma, active bleeding on contrast-enhanced CT seems to be the strongest predictor of urgent surgical intervention, whereas initial pleural drainage volume alone is not. Conservative management is safe in selected patients, while comorbidities influence postoperative outcomes. Multidisciplinary management and accurate radiological assessment are essential to guide timely and appropriate treatment.

## 1. Introduction

Traumatic hemothorax is a frequent consequence of blunt thoracic trauma and remains a significant cause of morbidity and mortality despite advances in trauma management. It results from the accumulation of blood within the pleural space, originating from the chest wall, lung parenchyma, intrathoracic vessels, or mediastinal structures, and may present with a clinical spectrum ranging from small, self-limiting collections to potentially life-threatening hemorrhage requiring urgent intervention [1].

Contrast-enhanced computed tomography (CT) currently represents the cornerstone of diagnosis and assessment of traumatic hemothorax, allowing accurate identification of pleural blood collections, associated thoracic injuries, and radiological signs of active bleeding [2].

Although the severity of hemothorax is often estimated on the basis of radiological findings and clinical parameters, the optimal criteria for selecting patients who require urgent chest drainage, angiographic embolization, or surgical exploration have not been fully defined, yet [3].

The currently available therapeutic strategies for traumatic hemothorax include clinical observation, chest tube insertion, transcatheter arterial embolization, and surgical treatment, either via video-assisted thoracoscopic surgery (VATS) or thoracotomy. Although chest tube placement represents the first-line treatment for patients with moderate-to-severe hemothorax or hemodynamic instability, a considerable proportion of patients require subsequent therapeutic escalation. Conversely, some patients can be safely managed with a conservative approach, highlighting the need to identify reliable predictors that can guide early decision-making [4,5].

Several studies have attempted to correlate the volume of hemothorax, clinical severity, and associated thoracic injuries with the need for invasive treatment. However, the relative weight of radiological findings, particularly the presence of active bleeding on contrast-enhanced CT, compared to clinical and laboratory parameters, remains a matter of debate. Furthermore, data on outcomes associated with different therapeutic strategies, including postoperative complications, length of hospital stay, and intensive care unit admission, are heterogeneous and often derived from limited or highly selected case series [6,7].

Therefore, this study aimed to evaluate the management of hemothorax resulting from blunt chest trauma at our center and to assess the associated clinical outcomes. Additionally, we analyzed risk factors associated with the need for urgent surgical intervention and the development of postoperative complications.

## 2. Materials and Methods

### 2.1. Study Design

This is a retrospective, single-center cohort study, conducted between January 2019 and December 2024 on patients admitted at our II Level Emergency Department (ED) with a diagnosis of hemothorax in blunt chest trauma. The study was approved by the Institutional Review Board (Università Cattolica del Sacro Cuore Approval Code: ID8098; Approval Date: 23 October 2025) and conducted in accordance with the principles of the Declaration of Helsinki. Data collection and reporting adhered to the Strengthening the Reporting of Observational Studies in Epidemiology (STROBE) guidelines.

The inclusion criteria were: adult patients (age ≥ 18 years old) with a clinical–radiological diagnosis of hemothorax in blunt chest trauma.

The exclusion criteria were: post-surgical bleeding, neoplastic or spontaneous hemothorax in patients in chronic anticoagulant therapy, and patients with stab or gunshot wounds.

Traumas were categorized according to the injury mechanism into traffic accidents, slip injuries, falls, crushes, etc. The AIS (Abbreviated Injury Scale) for chest trauma and the ISS (Injury Severity Score) for overall trauma, which were also extended to other districts, were also adopted to classify patients with scores below 16 and 16 or higher, a cut-off value that represents a severe, life-threatening condition, often correlating with a higher risk of mortality [8].

### 2.2. Italian Emergency Department System and Trauma Network

In our country, emergency care is organized within a regionalized network coordinated by the national emergency medical service (EMS, number “118/112”). Hospitals are structured into different levels based on available specialties, technological resources, and capacity to manage complex emergencies.

Level I EDs are equipped to manage most acute medical and surgical emergencies. They provide 24/7 availability of core specialties such as general surgery, internal medicine, orthopedics, anesthesiology, cardiology, and basic intensive care. They are capable of stabilizing Major Trauma patients but may transfer highly complex cases to higher-level centers when necessary.

Level II EDs represent tertiary referral centers within the regional network. In addition to all Level I capabilities, they provide advanced subspecialty services (e.g., neurosurgery, vascular surgery, interventional radiology, and cardiac surgery in some regions), comprehensive intensive care units, and advanced diagnostic resources.

In particular, for trauma, since 2015, a Ministerial Decree (DM 70/2015) has activated regional Major Trauma networks. The SIAT system (Integrated System for Trauma Assistance) used in several Italian regions (such as in Lazio, where our hospital is located) organized the trauma network in “hubs” and “spokes” integrated with the ED system for emergencies.

Level II centers often function as “hub” hospitals and may be designated as Highly Specialized Trauma Centers (HSTCs), receiving the most severe polytrauma cases directly from the scene or via secondary transfer.

The SIAT guarantees the continuity of care for traumatic emergencies through a three-level system:

− Level I or Emergency Departments for Trauma (EDT), that correspond to Level I EDs, are able to warrant ABC stabilization, basic diagnostic and treatment procedures, and, eventually, transfers to higher-level centers if needed (located in twenty-seven hospitals in the Lazio region);
− Level II or Area Trauma Centers (ATCs), that correspond to Level I EDs, are able to treat all trauma injuries except for those that require the highest specialization;
− Level III or Highly Specialized Trauma Centers (HSTCs), which represent level II EDs, with highly specialized facilities (i.e., thoracic surgery, neurosurgery, interventional radiology and maxillofacial surgery), are able to treat the most complex cases (three hubs located in Rome for the Lazio Region).

Within the Italian trauma system, patients are triaged pre-hospitally according to severity criteria and transported directly, when feasible, to the most appropriate facility (hub-and-spoke model). This organization directly influences patient case mix, timing of interventions, availability of advanced treatments, and therapeutic workflows.

In particular, Level II EDs concentrate higher clinical complexity, multidisciplinary management, and advanced procedural interventions, which have implications for both treatment strategies and data interpretation.

Our university hospital is the second largest hospital in Italy and the first in its region. It is a teaching hospital and one of the three hubs in the Lazio Region to which the spokes (ATCs and EDTs) refer in case of complexity. Since 2016, a Major Trauma Clinical Pathway (MTCP) has been established in our center with the intent of granting a continuum of effective care from the patients’ admission to the ED right up to their discharge and rehabilitation.

### 2.3. Diagnostic Pathway and Multidisciplinary Management

At our center, the trauma team leader is an intensivist physician. The trauma team is made up of a trauma and emergency surgeon, trauma orthopedic surgeon, trauma nurses and radiology staff [8,9,10]. Before the arrival of the patient by ambulance or helicopter, the pre-hospital emergency team alerts the trauma leader of the hub center by calling him at a dedicated phone number and giving information about the trauma dynamic and the patient’s condition and parameters.

Patients with blunt chest trauma and suspected hemothorax are managed in the emergency department according to a multidisciplinary approach, in accordance with ATLS guidelines [8,9]. Upon admission, patients are evaluated by the multidisciplinary trauma team.

Airway and hemodynamic stabilization, as well as potential ventilatory support, are managed by anesthesiologists, while diagnostic imaging—including FAST (Focused Assessment with Sonography for Trauma), eFAST (extended FAST for the detection of pneumothorax or hemothorax), Thorax X-ray, and contrast-enhanced computed tomography (CT) to identify signs of active bleeding from intercostal arteries, pulmonary vessels or parenchyma—is performed.

Thoracic surgeons are involved in assessing the indication for chest tube placement or urgent surgical intervention, particularly in cases of persistent bleeding, hemodynamic instability, severe radiological findings (such as vascular lesions, bleedings not suitable for embolization) or retained hemothax, according to ATLS recommendations [8].

Patient comorbidity burden is assessed using the Charlson Comorbidity Index (CCI), a validated tool for risk stratification and prognostic evaluation [11,12,13]. The CCI is used to support clinical decision-making and to guide the choice of the most appropriate management strategy [14,15].

### 2.4. Non-Surgical Treatment

In patients presenting with massive hemothorax (estimated volume of blood more than 1000/1500 cc on radiological imaging [3,8]), chest tube placement was performed.

All chest tubes (24 or 28 Fr) were inserted by experienced medical staff under sterile conditions at the fifth intercostal space along the mid-axillary line, close to the upper margin of the sixth rib, in order to minimize the risk of intercostal artery injury. Following insertion, all chest tubes were immediately connected to a one-way valve system to ensure adequate drainage of blood from the pleural cavity.

In cases where active bleeding from intercostal vessels was detected on CT scan, urgent angiography for potential transcatheter arterial embolization (TAE) was indicated.

TAE was performed via percutaneous femoral access and angiographic guidance for controlling hemorrhage from identified bleeding vessels [16,17].

Patients requiring ventilatory support were admitted to the intensive care unit, where invasive or non-invasive mechanical ventilation was provided according to clinical indications.

A conservative management with careful clinical–radiological observation was attempted in all those cases where clinical stability and negative CT for active bleeding, with an estimated amount of pleural effusion less than 1000 cc, were confirmed.

### 2.5. Surgical Treatment

**Figure 1:**
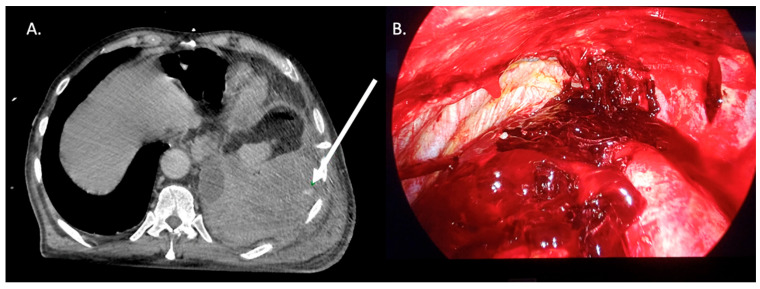
Left hemothorax due to intercostal artery bleeding in chest trauma. (**A**) CT scan showing the active bleeding (white arrow) and clots in the pleural space. (**B**) Intraoperative findings during VATS approach.

The decision to immediately proceed with surgical intervention was based on clinical criteria, such as hemodynamic instability, severe anemization and persistent blood output from the chest tube (200 mL/h for the next 2–4 h or more than 500 mL in the hour after chest tube placement), or hemothorax with active bleeding documented on CT scan (Figure 1) not suitable for embolization [18]. Surgical intervention was also indicated in case of retained hemothorax after chest tube placement or successful TEA.

All surgical procedures were performed under general anesthesia with single-lung ventilation. For exploratory VATS, a minimally invasive uniportal access was used, typically at the fifth intercostal space along the mid-axillary line, using a 30° 10 mm camera and long curve-shaped dedicated instruments.

For open procedures, a muscle-sparing lateral thoracotomy in the V intercostal space was performed.

The choice of surgical approach was guided by pre- and intraoperative hemodynamic status and the type of injury to be managed: the open approach was preferred over minimally invasive techniques only in selected cases of hemodynamic instability, in order to achieve faster and more effective control of any active bleeding or in case of severe organ (heart or lung) ruptures not easily manageable by VATS in emergency situation [19].

### 2.6. Outcomes

The primary outcomes of the study were to evaluate the number of patients who required an urgent placement of a chest tube or thoracic surgery and the type of surgery required. Secondary outcomes were: evaluating postoperative complications, hospitalization, length of chest tube and exploring any risk factor associated with the necessity of surgery and complications after surgery.

### 2.7. Statistical Analysis

Continuous variables are presented as mean ± standard deviation (SD) when normally distributed, or as median and interquartile range (IQR) in the case of non-normal distributions. The Shapiro–Wilk test was used to assess data normality. Differences between continuous variables were analyzed using the independent-samples Student’s *t* test, applying correction for unequal variances when necessary, or the Mann–Whitney U test for non-normally distributed data. Categorical variables are reported as frequencies and percentages and were compared using the chi-square test. Univariable and multivariable regression analyses were conducted to identify factors associated with surgical intervention and postoperative complications. Variables showing a *p* value < 0.20 in univariable analysis were entered into the multivariable model.

All statistical analyses were two-tailed, and a *p* value < 0.05 was considered statistically significant. Statistical processing was performed using IBM SPSS Statistics for Macintosh, version 25.0 (IBM Corp., Armonk, NY, USA).

Statistical analyses performed using IBM SPSS Statistics for Macintosh, version 25.0 (IBM Corp., Armonk, NY, USA).

## 3. Results

Among 378 patients admitted to our department with blunt chest trauma during the study period, only 72 (19.05%) had a diagnosis of traumatic hemothorax. Fifty-eight (80.6%) were male, with a mean age of 60.00 ± 20.47 years. The emergency codes assigned at triage were: high urgency in 58 cases (80.6%), moderate in 11 (15.3%) and low urgency in 3 (4.1%).

The main clinical characteristics and admission parameters of the patients analyzed are reported in Table 1.

According to the AIS score for thoracic trauma, 59 patients (81.9%) were classified as AIS = 3 (serious thoracic trauma) and 13 (18.1%) as AIS = 4 (severe trauma). On the basis of injuries extended to other organs (mainly extremities and abdomen in our series), the ISS score was the following: in 51 traumas (70.8%), the ISS score was <16 (only AIS = 3 chest trauma), and in 21 cases (29.2%), the ISS was ≥16 (13 cases with ISS = 16 (all chest traumas with AIS = 4), 6 cases with ISS = 18 (AIS = 3 for chest and AIS = 3 for extremities), 2 cases with ISS = 25 (both AIS = 3 for chest and one AIS = 4 for abdomen, and the other one AIS = 4 for extremities, respectively)).

No difference (*p* = 0.412) was recorded between the different trauma mechanisms in terms of trauma severity (ISS ≥ 16) in our series.

In 61.1% of cases (44 patients), the primary cause of hemothorax was rib fractures, followed by lung contusion or laceration (11 cases, 15.3%) and by multiple associated injuries involving the ribs, lung, or diaphragm (11 cases, 15.3%). The severity of hemothorax detected on contrast-enhanced emergency CT scan or the presence of hemodynamic instability required chest tube placement (thoracostomy) in 51 patients (70.8%). The mean volume of blood drained immediately after chest tube insertion was 1274.71 ± 842.81 mL.

In 12 cases (16.7%), including 9 (12.5%) that had already been drained before CT, active bleeding from intercostal arteries or lung parenchyma was identified on CT scan, prompting urgent angiography. TEA was successfully performed in seven patients (9.7%).

Nine patients (12.5%) required immediate orotracheal intubation and invasive mechanical ventilation at the emergency department admission or during diagnostic examinations.

In 23 cases (31.9%), urgent surgical intervention was indicated by the thoracic surgeon based on hemodynamic status, persistent blood output from chest drainage, CT findings, unsuccessful TEA or retained hemothorax. Among these patients, 16 (22.2%) underwent chest tube placement before surgical indication, while 7 (9.7%) were referred to urgent surgery directly.

However, in the case of previous chest tube placement, no significant difference in blood volume drained immediately after chest tube insertion was observed between patients who underwent urgent surgery and those managed non-operatively (1242.86 ± 908.87 mL vs. 1312.50 ± 701.31 mL, *p* = 0.787).

Interestingly, no correlation was found between an ISS score ≥ 16 and the presence, or not, of active bleeding on a CT scan (4 vs. 16, *p* = 0.638), the need or absence of chest tube placement (11 vs. 9, *p* = 0.07) or the requirement, or not, for urgent thoracic surgery (7 vs. 13, *p* = 0.730).

Surgical management was performed via VATS approach in 10 cases to explore the pleural cavity, control bleeding sources, and evacuate clots. Lung resection was required in three patients due to extensive parenchymal destruction and active bleeding. Intraoperative identification of active bleeding occurred in only six cases.

Multivariable analysis identified active bleeding on CT scan as the only independent risk factor for urgent surgery (OR = 3.850, 95% CI 1.068–13.880, *p* = 0.039), as shown in Table 2.

Fourteen patients (19.4%) required neither chest drainage nor surgical intervention and were managed conservatively in medical wards.

Overall, 29 patients (40.3%), including those who underwent surgery, required admission to the intensive care unit for at least one day following the trauma, after which all were transferred to a surgical ward.

Postoperative complications occurred in five patients, including two cases of respiratory failure, one case of pneumonia, and two cardiac complications. During multivariable analysis, only the CCI showed a trend toward significance as an independent risk factor for postoperative complications (OR = 5.856, 95% CI 0.823–41.668, *p* = 0.077), as shown in Table 3.

The surgical chest tube was removed after a median of 4 days, whereas thoracostomy tubes in non-surgical patients were removed after a median of 5 days.

The overall median length of hospital stay was 11 days and was significantly shorter in patients admitted to surgical wards compared with those managed in medical wards (10.15 ± 7.75 vs. 19.71 ± 12.44 days, *p* = 0.001). This finding may be explained by the significantly higher proportion of patients managed non-surgically compared to those treated surgically, who were admitted to medical wards (11 vs. 1, *p* = 0.05), and by a higher mean CCI that affects patients assigned to medical wards (3.50 ± 2.51 vs. 1.80 ± 1.75, *p* = 0.006).

Overall mortality was 5.6% (four patients), attributable to severe respiratory complications and multi-organ failure. Thirty-day readmission occurred in two patients (2.9%), both due to retained hemothorax following removal of the thoracostomy tube. No correlation between an ISS score ≥ 16 and mortality (*p* = 0.370) or 30-day readmission (*p* = 0.381) was also recorded.

## 4. Discussion

In this retrospective study conducted in a Level II ED, we analyzed a consecutive cohort of 72 patients with traumatic hemothorax resulting from blunt chest trauma, aiming to evaluate clinical and surgical management and to identify factors associated with the need for urgent invasive intervention. The prevalence of hemothorax observed in our center (19.05% of patients with blunt chest trauma) is consistent with the literature, confirming that hemothorax represents one of the most frequent and potentially severe complications of chest trauma [1].

The initial management of these patients plays a crucial role, as inadequate treatment in the emergency setting may lead to severe consequences, including respiratory failure and distress, hypovolemic shock, lung compression, and, in the most severe cases, death. In this context, several studies have demonstrated that the structured involvement of multiple specialties and organizations within regionalized trauma systems is associated with a significant reduction in mortality [20,21,22,23].

An adequate team-based approach, with a horizontal distribution of responsibilities among the different professionals involved, is considered an essential element for the appropriate management of the trauma patient [8,9,10].

At our center, in accordance with ATLS guidelines [8] and the national SIAT system, patients with chest trauma are evaluated upon arrival in the II Level ED by a multidisciplinary team, generally coordinated by an emergency physician and a surgeon.

In our cohort, rib fractures represented the main cause of hemothorax (61.1%), confirming that chest wall injury is the most frequent mechanism of bleeding in blunt trauma. However, although the severity of trauma (ISS score) was also evaluated among potential factors associated with the risk of urgent thoracic surgery, the most relevant finding emerged from the multivariable analysis as an independent factor associated with the need for urgent surgical intervention [2] was the presence of active bleeding on a contrast-enhanced CT scan (OR = 3.850) rather than the severity of trauma itself.

This finding suggests that, within a comparable clinical context, the volume of hemothorax at a CT scan per se does not represent the main determinant of therapeutic decision-making, whereas radiological or clinical (persistent blood output from chest drain in the following hours after placement) evidence of ongoing hemorrhage plays a decisive role [3].

Supporting this interpretation, in our study, no significant differences (*p* = 0.787) were observed in the volume of blood drained immediately after chest tube insertion between patients who underwent surgery and those who did not. This finding also underscores the limited value of initial pleural drainage volume alone as a predictor for therapeutic escalation, compared with evidence of ongoing active bleeding.

These results are consistent with the literature, which emphasizes that although volumetric criteria are widely used in clinical practice, they are characterized by high heterogeneity and the absence of universally accepted thresholds.

It is noteworthy that active bleeding was documented intraoperatively in only a portion of the surgically treated patients, despite its presence on preoperative CT scans. In the context of traumatic hemothorax, it is not uncommon that no active bleeding source is identified at the time of pleural cavity exploration. This phenomenon is particularly observed in cases of pulmonary parenchymal injury and described in thoracic trauma literature. Small parenchymal bleeding visualized on contrast-enhanced CT may represent transient vascular extravasation that subsequently decreases or ceases due to intrinsic tamponade mechanisms, including lung recoil, local clot formation, low-pressure pulmonary circulation, and the compressive effect of accumulating hemothorax [24]. As a result, active bleeding seen radiologically may no longer be evident intraoperatively a few hours later.

Therefore, the absence of active bleeding at surgery does not necessarily indicate overtreatment or selection bias, but instead may reflect the natural evolution of traumatic parenchymal hemorrhage between imaging and operative intervention. In fact, CT evidence of active bleeding remains clinically relevant because it identifies patients at high risk for ongoing or recurrent hemorrhage, justifying early surgical or interventional management. This finding highlights the need for a decision-making process that integrates clinical, hemodynamic, and radiological data rather than relying on a single parameter.

A significant proportion of patients (19.4%) were managed without the need for chest tube placement or surgical intervention. This confirms that, in selected and clinically stable patients, observation can be a safe strategy. However, this approach requires close monitoring and immediate availability of therapeutic escalation, particularly in the presence of worsening clinical condition [17].

At our center, the assessment of comorbidity burden using the CCI served as an additional tool to guide the clinical–surgical strategy. The CCI, introduced in 1987 to estimate one-year mortality risk in hospitalized patients, allows summarizing the prognostic impact of 19 comorbid conditions into a single score [11,12,13]. Currently, the CCI remains a widely used tool for risk stratification and the personalization of clinical decision-making [15].

In our study, the CCI showed a trend toward an independent association with the development of postoperative complications (OR = 5.856; *p* = 0.077). Although the result may be affected by the limited sample size that weakens the robustness of the multivariable model, it suggests that the comorbidity burden may influence surgical tolerance and the risk of adverse events, particularly respiratory and cardiovascular.

Another notable finding from our cohort concerns the length of hospital stay. The mean duration of hospitalization was significantly shorter for patients managed in surgical wards compared to those in medical wards (10.15 ± 7.75 vs. 19.71 ± 12.44 days; *p* = 0.001). This result may reflect, on one hand, the earlier and more structured management in patients undergoing invasive treatment, with a faster therapeutic pathway and intensive initial monitoring, followed by rapid transfer and discharge. Conversely, conservatively managed patients may require a longer period of observation and monitoring, especially if they have significant comorbidities or secondary respiratory complications. On the other hand, the longer hospitalization recorded in medical wards reflects the consequences of a significantly higher mean CCI score of patients assigned to these wards. Therefore, this result should be interpreted with caution, as the difference could be influenced by either selection factors or different management among wards.

The overall mortality rate of 5.6% observed in our cohort is consistent with the literature on traumatic hemothorax and further underscores the importance of early, structured, and multidisciplinary management of these patients [25,26].

Overall, the results of our study support the hypothesis that the presence of active bleeding on contrast-enhanced CT represents a predictor of the need for urgent surgical intervention. This finding has practical implications: in patients with traumatic hemothorax, CT findings should guide the decision-making process, prompting a more aggressive and timely management approach in case of active bleeding, even in the absence of a large-volume pleural drainage. Furthermore, the assessment of comorbidities using the CCI can help identify patients at higher risk of postoperative complications and allow for the planning of a more protective care pathway.

### Limitations and Point of Strengths

This study has some inherent limitations related to its retrospective and single-center design that can limit the generalizability of the results. Moreover, the relatively small cohort of patients, the national-specific pathway for trauma network and the possible heterogeneity of clinical decision-making criteria may prevent the applicability of these findings beyond the study cohort. Also, the lack of pre-hospital data in our series, due to the absence of complete integration between pre-hospital systems and hospital data in the whole nation (Italy’s healthcare system is highly decentralized, with each region responsible for its own systems), prevents us from having a more data-driven, patient-centric analysis. Additionally, the assessment of active bleeding on CT was based on radiology reports and not on a centralized review, which could introduce inter-observer variability. Furthermore, aside from the total volume of blood drained immediately after chest tube placement, no data were available on potential ongoing bleeding from the chest tube or on the volume of drainage in the subsequent hours.

This finding may have led to urgent surgical indications in real-world practice; however, its absence in the available data analyzed may represent a limitation to be considered when interpreting the results. The blood gas analysis parameters were also not retrievable, and therefore their potential impact was not evaluated. On the other hand, the main strength of the study lies in the consecutive patient cohort from a high-volume Level II ED and a multidisciplinary approach in the management of each case.

## 5. Conclusions

In our series, first, the presence of active bleeding on a contrast-enhanced CT scan seems to be the only independent factor associated with the need for urgent surgical intervention in patients with traumatic hemothorax from blunt chest trauma. Second, the immediate volume of pleural blood output documented by chest tube drainage alone was not predictive of therapeutic escalation. Third, conservative management was safe in a selected subset of patients, while postoperative complications were primarily related to the burden of comorbidities.

In conclusion, these findings highlight the importance of a multidisciplinary approach and accurate radiological assessment to guide therapeutic decisions and optimize outcomes. In this context, the availability of an organized and standardized pathway, based on guidelines and shared radiological and clinical criteria, can improve the quality of care and patient outcomes.

## Figures and Tables

**Table 1:** Clinical characteristics and outcomes of patients analyzed.

	Patients (n = 72)
**Mean age (years)**	60.00 ± 20.47
**Male sex**	58 (80.6%)
**Comorbidities: Atrial fibrillation**	5 (6.9%)
** Heart failure**	2 (2.8%)
** History of cardiac infarction**	2 (2.8%)
** Peripheral vasculopathy**	1 (1.4%)
** History of stroke/TIA**	4 (5.6%)
** Dementia**	3 (4.2%)
** COPD**	4 (5.6%)
** Connective tissue disorders**	-
** Liver disease**	1 (1.4%)
** Cirrhosis**	1 (1.4%)
** Diabetes**	2 (2.8%)
** Renal failure**	-
** Malignancies**	4 (5.6%)
**Charlson score: 0**	23 (31.9%)
** 1**	13 (18.1%)
** 2**	5 (6.9%)
** 3**	11 (15.3%)
** 4**	11 (15.3%)
** 5**	6 (8.3%)
** 6**	1 (1.4%)
** 7**	2 (2.8%)
**Trauma mechanism: Traffic accidents**	25 (34.7%)
** Slip injuries**	24 (33.3%)
** Falls**	14 (19.5%)
** Crushes **	9 (12.5%)
**Diagnosis: Rib fractures**	44 (61.1%)
** Sternum fracture**	4 (5.6%)
** Diaphragmatic rupture**	2 (2.7%)
** Pulmonary laceration**	11 (15.3%)
** Cardiac rupture**	-
** More than one lesion**	11 (15.3%)
**CT signs of active bleeding**	12 (16.7%)
**Glasgow Coma Scale**	13.81 ± 3.73
**Min blood pressure (mmHg)**	80.29 ± 16.19
**SpO_2_ in air**	92.76 ± 3.62
**Hemoglobin value (g/dL)**	12.20 ± 2.30
**Chest tube insertion**	51 (70.8%)
**Thoracic surgery**	23 (31.9%)
**Thoracic approach: VATS**	10 (13.9%)
** Thoracotomy**	13 (18.1%)
**Intraoperative signs of active bleeding**	6 (8.3%)
**Other associated surgeries: Orthopedic surgery**	7 (9.7%)
** Neurosurgery**	0 (0%)
** Cardiac surgery**	0 (0%)
** Abdominal surgery**	1 (1.4%)
** Urology**	0 (0%)
**Postoperative complications: **	
** Respiratory failure**	2 (2.8%)
** Pneumonia**	1 (1.4%)
** complications**	0 (0%)
** Other**	2 (2.8%)
**Surgical ward admission**	50 (69.4%)
**Thoracic surgery ward admission**	35 (48.6%)
**ICU admission**	29 (40.3%)
**Mechanical ventilation**	9 (12.5%)

**Table 2:** Univariable and multivariable analyses of risk factors associated with urgent surgery (significant values in bold, *p* < 0.05).

	Univariable Analysis	Multivariable Analysis	
	*p*-Value	OR [95% CI]	*p*-Value
**Male sex**	0.114		
**Age**	0.557		
**Charlson Comorbidity Index**	0.697		
**Chest tube placement**	0.871		
**Active blush on CT scan**	**0.032**	3.850 [1.068–13.880]	**0.039**
**Angiography**	0.132		
**Blood output chest drain**	0.787		
**Injury Severity Score** ≥ **16**	0.730		
**Heart rate**	0.578		
**Min blood pressure**	0.680		
**Hemoglobin value**	0.303		

**Table 3:** Univariable and multivariable analyses of risk factors associated with postoperative complications (significant values in bold, *p* < 0.05).

	Univariable Analysis	Multivariable Analysis	
	*p*-Value	OR [95% CI]	*p*-Value
**Male sex**	0.290		
**Age**	0.425		
**Charlson Comorbidity Index**	0.078	5.856 [0.823–41.668]	0.077
**Chest tube placement**	0.074		
**Invasive ventilation**	0.658		
**Thoracotomy access**	0.193		
**Other associated surgeries**	**0.033**	10.716 [0.525–218.813]	0.123
**Injury Severity Score** ≥ **16**	**0.925**		
**Hospital stay**	0.443		

## Data Availability

The datasets generated and/or analyzed during the current study are available from the corresponding author on reasonable request.
